# Supplementary material for: Influence of Different Salts on the G-Quadruplex Structure Formed from the Reversed Human Telomeric DNA Sequence
Source: Int J Mol Sci. 2022 Oct 13;23(20):12206. doi: 10.3390/ijms232012206 (PMC9602856; doi:10.3390/ijms232012206)
Supplement: Supplementary file 1 [file ijms-23-12206-s001.zip › ijms-1891068-supplementary.pdf]

Supporting Information

Influence of different salts on the G-quadruplex structure formed from the reversed human telomeric DNA sequence

Lydia Olejko,[†] Anushree Dutta,[†] Kosar Shahsavar,[‡] Ilko Bald*

Institute of Chemistry – Hybrid Nanostructures, University of Potsdam, Karl-Liebknecht-Str. 24-25, 14476 Potsdam, Germany

‡ These authors contributed equally.

‡ permanent address: Department of Life Science Engineering, Faculty of New Sciences & Technologies, University of Tehran, Tehran, Iran

*corresponding author: ilko.bald@uni-potsdam.de

<u>CD spectroscopy for HumTel</u>

The human telomeric DNA ((TTA GGG)$_n$) is known for its structural polymorphism. Here, we have analyzed TT (GGG TTA)$_3$ GGG TTT with CD spectroscopy. As shown in Figure S1 the CD spectrum of the folded G-quadruplex changes in presence of different salts (KCl, NaCl, $MgCl_2$, $CaCl_2$). Hence, the specific G-quadruplex structures is influenced by the different cations.

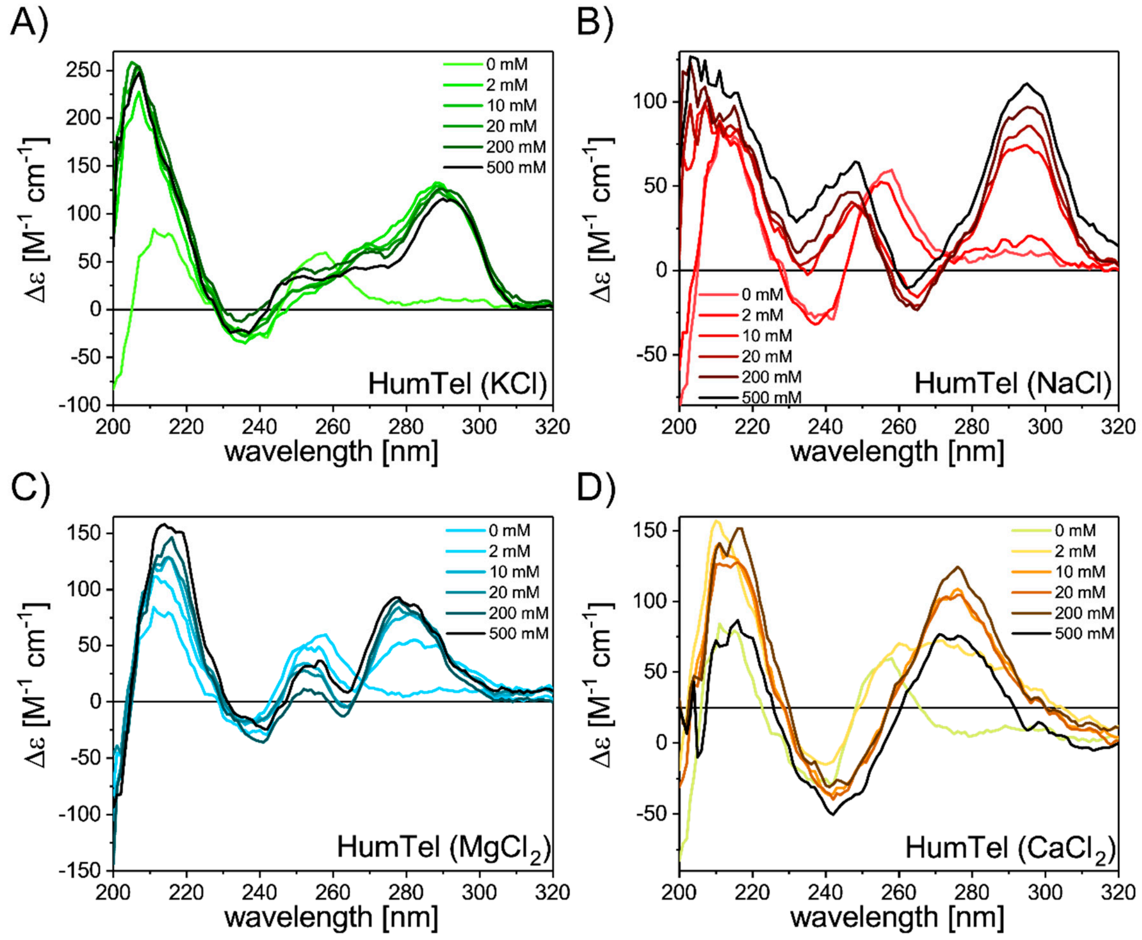

Figure S1. CD spectra of HumTel in presence of different salts (KCl (A), NaCl (B), $MgCl_2$ (C) and $CaCl_2$ (D)). The salt concentration ranges from 0 mM (light color) to 500 mM (dark color). The CD spectrum is influenced in presence of different salt meaning that the G-quadruplex structure changes in presence of different cations.

Influence of the buffer

The TAE buffer has no influence on the CD spectrum when compared to the free telomeric DNA diluted in ultra-pure water (see Figure S2).

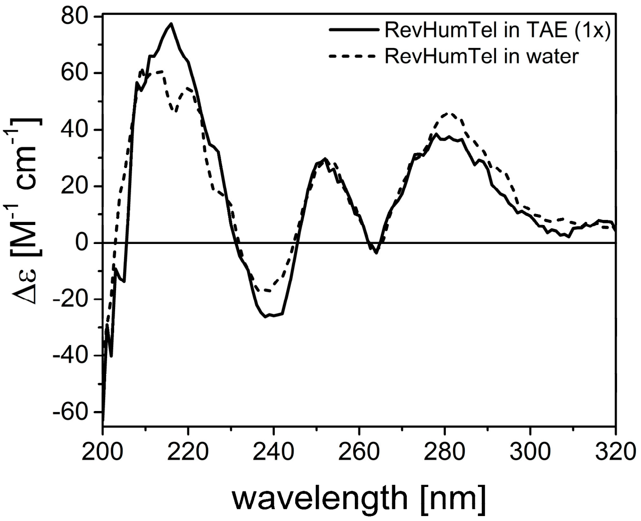

Figure S2. CD spectra of RevHumTel in TAE buffer (solid) and ultra-pure water (dashed). The CD spectrum is not influenced by the buffer.

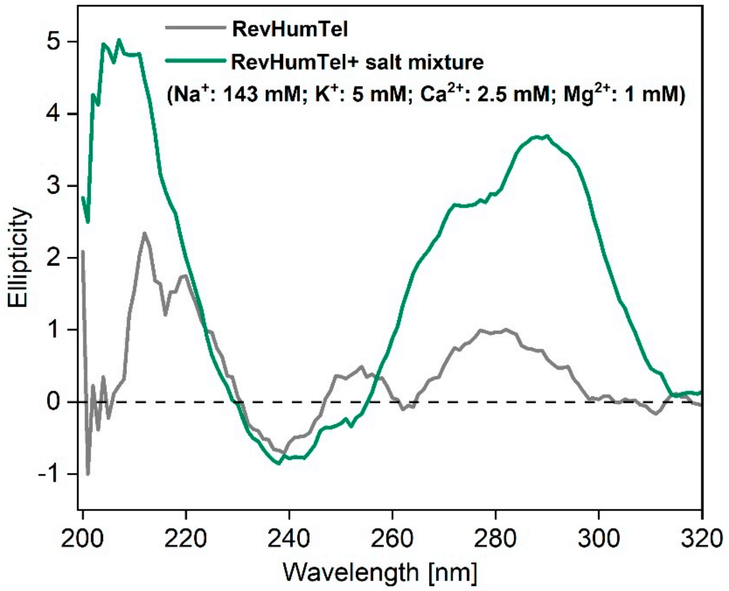

Figure S3. CD spectra of RevHumTel (c = 8.6 µM) only and in presence of mixture of salts showing the dominance of hybrid-type G-quadruplex structure.

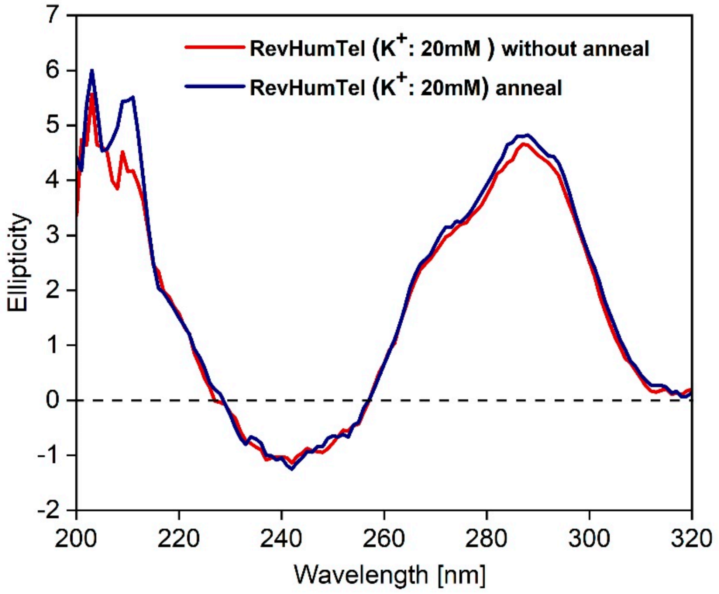

Figure S4. CD spectra of RevHumTel (c = 8.6 µM) in presence of KCl (20mM) recorded after incubation for 15 min at RT (red curve) and after annealing for 15 min at 40 °C (blue).

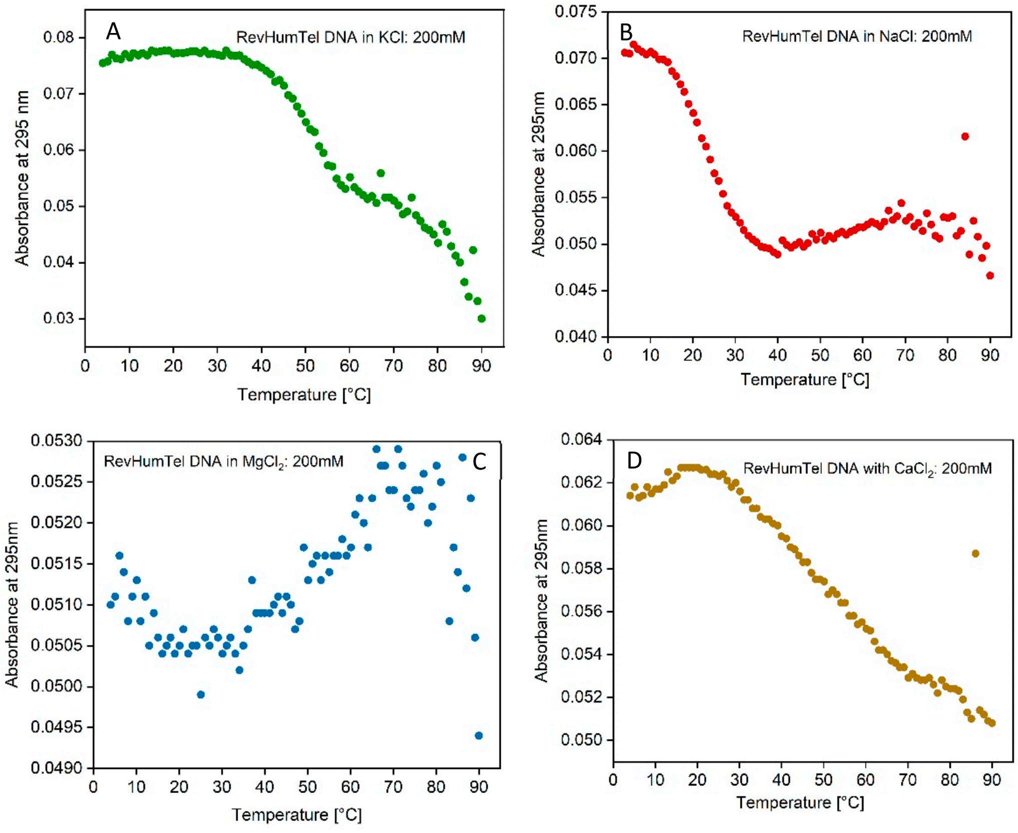

Figure S5. UV-thermal melting curves of RevHumTel in presence of different salts (A) KCl, (B) NaCl, (B) $MgCl_2$, and (D) $CaCl_2$. For all salt mixture, a final concentration of 200 mM is maintained. A biphasic melting profile in case of RevHumTel-KCl and NaCl mixture is observed (with melting points at 50 °C and 80 °C for KCl, and 23 °C and 81 °C for NaCl). For $MgCl_2$ no clear melting behaviour could be observed, while $CaCl_2$ gives rise to a broad melting stage with a melting temperature of 45 °C, suggesting that multiple conformations are present with close melting temperatures.

Spectral overlap of Fluorescein and Cy3

The software PhotochemCAD 2.1 has been used to calculate the spectral overlap integrals *J* and the Förster radius R_0 for FAM and Cy3. The FAM emission and Cy3 absorption spectra (acceptor's absorption spectrum in terms of extinction coefficient) are imported and the dipole orientation factor (κ_2 = 2/3), the refractive index (n = 1.33) and the donor's quantum yield (ϕ*(FAM)* = 0.90) are used as inputs in the software. The determined spectral overlap integral is $5.6 \cdot 10^{15}$ $nm^4 \cdot l \cdot mol^{-1}$ and the Förster radius for FAM/Cy3 is 6.7 nm. The donor's emission and acceptor absorption spectra are shown in Figure S6.

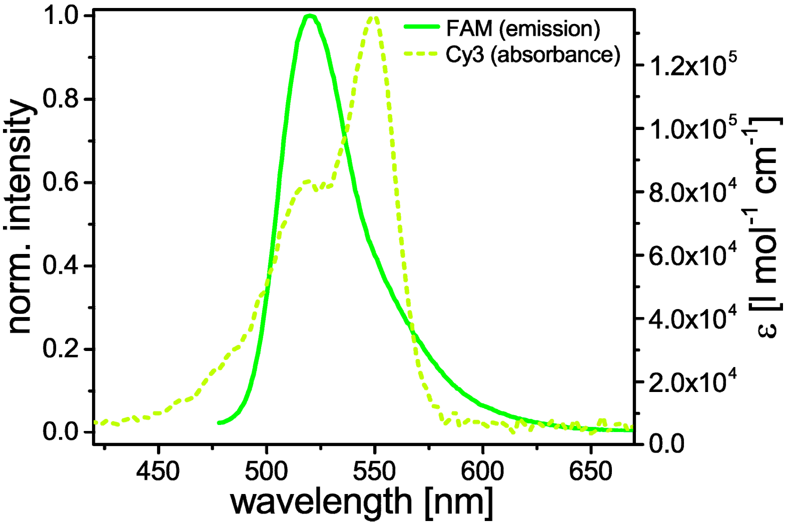

Figure S6. Spectral overlap of FAM emission (green) and Cy3 (yellow) absorption spectra.
